# On the trade-off between single-neuron complexity and network size with respect to spike-timed computations

**DOI:** 10.1186/1471-2202-13-S1-P185

**Published:** 2012-07-16

**Authors:** Venkatakrishnan Ramaswamy, Arunava Banerjee

**Affiliations:** 1 Interdisciplinary Center for Neural Computation, The Edmond and Lily Safra Center for Brain Sciences, Hebrew University, Jerusalem 91904, Israel; 2 Computer and Information Science and Engineering, University of Florida, Gainesville, FL 32611, USA

## 

Certain types of neurons in the brain seem to possess richer computational ability at the single-neuron level than others due to anatomical as well as physiological factors such as elaborate dendritic arbors and a larger variety of ion channels respectively. While, clearly, such features seem to endow the neurons themselves with a richer repertoire of computations, it is less clear how this exactly affects computation at the network level. For instance, is it true that there exist computations performed by large networks of “simple” neurons that can be achieved by small networks of more “complex” neurons (possessing such richer computational ability)? If so, what is the nature of the trade-off between the complexity of single neurons and the size of network required to achieve certain computations? In other words, can we quantify the number of “complex” neurons required to achieve the computation in question, given precise constraints on the computational ability of the “complex” neurons available?

In order to examine these questions, as a first step, we study here the case of feedforward networks of neurons. When a feedforward network is driven with input spike trains that start with quiescence, one can view the network as a transformation, which is to say a function that maps input spike trains to output spike trains (As a simplifying assumption, we do not treat stochastic variability in the response of neurons). In this setting, the computation performed by the network is exactly a transformation, as described above. Here, our main result, in essence, is that feedforward networks equipped with ``simple'' neurons, where the network may have an arbitrary number of neurons and arbitrary depth turn out to have small-sized equivalent networks of depth equal to two made up of more complex neurons that achieve *exactly* the same spike-train to spike-train transformation.

Our broad approach is to consider a large class of neuron models that include “complex” neuron models as well as “simple” neuron models. Technically speaking, this class of neuron models is characterized by the fact that the models in it satisfy certain properties. Each such model has a membrane potential function that depends on input spikes received and output spikes emitted by the neuron in a bounded past time-window, with the following constraints. (a) The neuron settles to the resting potential upon receiving no input spikes in the input time-window and having no output spikes in the output time-window, (b) The neuron has an absolute refractory period, (c) Relative refractory period effects, i.e. presence of spikes in the past output time-window has a hyperpolarizing effect vis-à-vis absence of spikes in the output time-window, when spikes in the input time-window are the same in either case; and (d) The neuron emits an output spike when its membrane potential hits a certain threshold. Notably, no specific functional form of the membrane potential is assumed for this class of neuron models. This class of neuron models includes, up to arbitrary accuracy, a number of contemporary neuron models such as the Leaky Integrate-and-Fire Model and the Spike Response Model, although models significantly more powerful satisfy the aforementioned properties as well. Our main technical result then states the following. Given a feedforward network (with arbitrary number of neurons and arbitrary depth), each of whose constituent neurons obey a model that lies in the aforementioned class, with the network having *n* output neurons, there exists an equivalent feedforward network of depth equal to two having *2n* neurons (each satisfying a model in the same class), so that the latter network effects *exactly* the same spike-train to spike-train transformation as the former. This, in particular, implies that arbitrarily large feedforward networks of “simple” neurons have very small equivalent networks of “complex” neurons.

These results provide an impetus to explore several questions in this direction. For instance, tailoring the class of neuron models considered to reflect specific anatomical and physiological features of complex biological neurons might yield better insights on the nature of this fundamental trade-off and help us understand why evolution may have chosen large networks of simple neurons for some tasks and small networks of complex neurons for others.

